# High-Salt Diet Induces IL-17-Dependent Gut Inflammation and Exacerbates Colitis in Mice

**DOI:** 10.3389/fimmu.2017.01969

**Published:** 2018-01-15

**Authors:** Sarah Leão Fiorini Aguiar, Mariana Camila Gonçalves Miranda, Mauro Andrade Freitas Guimarães, Helton Costa Santiago, Camila Pereira Queiroz, Pricila da Silva Cunha, Denise Carmona Cara, Giselle Foureaux, Anderson José Ferreira, Valbert Nascimento Cardoso, Patrícia Aparecida Barros, Tatiani Uceli Maioli, Ana Maria Caetano Faria

**Affiliations:** ^1^Departamento de Bioquímica e Imunologia, Instituto de Ciências Biológicas, Universidade Federal de Minas Gerais, Belo Horizonte, Brazil; ^2^Departamento de Morfologia, Instituto de Ciências Biológicas, Universidade Federal de Minas Gerais, Belo Horizonte, Brazil; ^3^Departamento de Análises Clínicas e Toxicológicas, Faculdade de Farmácia, Universidade Federal de Minas Gerais, Belo Horizonte, Brazil; ^4^Departamento de Nutrição, Escola de Enfermagem, Universidade Federal de Minas Gerais, Belo Horizonte, Brazil

**Keywords:** sodium chloride, gut inflammation, IL-17, Th17 cells, ILC3

## Abstract

Excess intake of sodium is often associated with high risk for cardiovascular disease. More recently, some studies on the effects of high-salt diets (HSDs) have also demonstrated that they are able to activate Th17 cells and increase severity of autoimmune diseases. The purpose of the present study was to evaluate the effects of a diet supplemented with NaCl in the colonic mucosa at steady state and during inflammation. We showed that consumption of HSD by mice triggered a gut inflammatory reaction associated with IL-23 production, recruitment of neutrophils, and increased frequency of the IL-17-producing type 3 innate lymphoid cells (ILC3) in the colon. Moreover, gut inflammation was not observed in IL-17–/– mice but it was present, although at lower grade, in RAG^−/−^ mice suggesting that the inflammatory effects of HSD was dependent on IL-17 but only partially on Th17 cells. Expression of SGK1, a kinase involved in sodium homeostasis, increased 90 min after ingestion of 50% NaCl solution and decreased 3 weeks after HSD consumption. Colitis induced by oral administration of either dextran sodium sulfate or 2,4,6-trinitrobenzenesulfonic acid was exacerbated by HSD consumption and this effect was associated with increased frequencies of RORγt^+^ CD4^+^ T cells and neutrophils in the colon. Therefore, our results demonstrated that consumption of HSD *per se* triggered a histologically detectable inflammation in the colon and also exacerbated chemically induced models of colitis in mice by a mechanism dependent on IL-17 production most likely by both ILC3 and Th17 cells.

## Introduction

Sodium is an indispensable nutrient for proper cell functions in living animals when consumed in appropriate amounts (1). It is essential for maintenance of osmotic pressure, normal pH, distribution of body fluids, and for most of metabolic processes. The influx of sodium ions across plasma membrane is required for action potential involved in nerve impulses and muscle contraction. Sodium chloride is readily available in processed food and ingested in large amounts as part of Western diets (2). Excess intake of sodium is often associated with high risk for cardiovascular diseases (2–4).

It has been reported recently that immune cells play an important role in mediating the detrimental effects associated with consumption of high concentration of sodium. Elevated concentration of sodium chloride was found to induce proinflammatory IL-17-producing helper T (Th17) cells *via* p38/MAPK/nuclear factor of activated T cells 5 pathway and the serum/glucocorticoid-regulated kinase 1/forkhead box protein 1 (SGK1) pathway both *in vitro* and *in vivo* (5, 6). These studies have demonstrated that high-salt diets (HSDs) exacerbate autoimmune encephalomyelitis in mice in a SGK1-dependent fashion (5, 6). In addition, hypertension and renal injury induced by HSD in salt-sensitive rats are accompanied by increased infiltration of T cells in kidneys (7, 8).

Intestinal mucosa is the major surface of contact with the external environment and the first tissue to interact with all types of food components including salt. The gastrointestinal tract is in constant interaction with the microbiota and the antigens from diet. Under normal conditions, exposure to these antigenic proteins would induce oral tolerance. However, any failure in intestinal homeostasis may result in inflammatory reactions toward microbiota and food antigens (9). These reactions are especially present during inflammatory bowel diseases (IBDs) such as Crohn’s disease (CD) and ulcerative colitis (UC). It has been proposed that IBD results from the imbalance between regulatory T cells (Treg) and inflammatory Th1, Th2, and Th17 cells (10). Several studies have demonstrated an increased expression of Th17-related cytokines in both UC and CD, although it is not clear whether these cytokines are only produced by T cells (11).

Dietary composition has been long seen as a potential risk factor for the increased incidence of autoimmune diseases and IBD. A specific factor that has changed in the Western diet over time is the increasing amount of sodium-containing salt intake. Some recent studies have demonstrated that diets containing high amounts of sodium chloride can also induce an increase in Th17 cells and aggravation of experimental models of colitis by the activation of SGK1 kinase (12–14). These studies highlighted the importance of SGK1/Th17 axis but did not explore appropriately the possibility that IL-17 could be produced by other sources such as ILC3. It is clear that several cell types other than lymphocytes are important in intestinal homeostasis and also in the onset of inflammatory diseases (15).

Innate lymphoid cells (ILCs) represent a heterogeneous group of hematopoietic cells of the innate immune system that have a common lymphoid progenitor. While ILCs lack rearranged antigen-specific receptors, they express many of the transcription factors and effector molecules expressed by CD4^+^ T helper (Th) cell populations, suggesting that ILCs may be an evolutionary precursor of cells of the adaptive immune system. They also have homologous functions with the Th cells. ILC1 express the transcription factor t-bet and is homologous to Th1 cells; ILC2 expresses GATA 3 and are homologous to Th-2 cells and ILC3 express ROR-γt and are homologous to Th-17 cells (16, 17). Considering that Th17 cells have been demonstrated to be pivot cells in pathogenicity of HSD in encephalomyelitis (5, 6) as well as colitis (12–14) models, that ILC3 are very abundant in the gut mucosa and that they are increased in the colon of individuals during the course of IBD (17, 18), it is likely that the effect of HSDs in colitis development is dependent on IL-17 production by both Th17 cells and ILC3.

This study aims to investigate the direct effect of a sodium chloride-rich diet in the gut mucosa as well as in the development of chemically induced colitis. Our working hypothesis is that sodium chloride may have an inflammatory effect *per se* and that both T cells and ILCs may play a role in this effect. In addition, we sought to investigate the synergistic effect of HSD in the inflammatory events taking place during experimental colitis induced by administration of 1% dextran sodium sulfate (DSS) in C57BL/6 mice and in the colitis induced by 2,4,6-trinitrobenzenesulfonic acid (TNBS) in BALB/c mice.

## Materials and Methods

### Animals

Female BALB/c and C57BL/6 mice were purchased from CeBio (Centro de Bioterismo da Universidade Federal de Minas Gerais). Eight-week-old animals were used. IL-17^−/−^ and RAG^−/−^ mice were kindly provided by Dr. João Santana from Universidade de São Paulo, Ribeirão Preto, Brazil, and by Dr. Ricardo Gazzinelli, Universidade Federal de Minas Gerais, Belo Horizonte, Brazil, and maintained in our animal facility located at Instituto de Ciências Biológicas, Universidade Federal de Minas Gerais. Animals were bred and housed in microisolators. All procedures were approved by the local ethical committee for animal research (CEUA-UFMG, Brazil, protocol 50/2014). Experiments were performed in accordance with guidelines and regulation established by Conselho Nacional de Controle de Experimentação Animal (CONCEA), Brazil.

### High-Salt Diet

Mice received either AIN93G diet as a control diet (15) or modified AIN93G supplemented with 4% NaCl (HSD) throughout the experiments.

### Hemodynamic Measurements Using the Tail-Cuff Method

Mean arterial pressure (MAP) was evaluated by a volume pressure recording sensor and an occlusion tail-cuff, which measures mice’s tail blood pressure noninvasively (Kent Scientific Corporation, Torington, CT, United States) (16). Mice were acclimated to restraint and tail-cuff inflation for 2 days before the beginning of experiments. The restraint platform was maintained at 31–34°C. In each session, mice were placed in an acrylic retainer, and the tail was inserted into a compression cuff that measured the blood pressure eight times. Following the measurement cycle, the average of these values was considered for each mouse. All parameters were evaluated weekly, 1 day before the beginning of dietary consumption as well as 7, 14, and 21 days thereafter.

### Intestinal Permeability Test

Intestinal permeability was determined by measuring radioactivity diffusion in the blood after oral administration of diethylenetriamine pentaacetic acid (DTPA) labeled with ^99m^-technetium (^99m^Tc). After 21 days of diet consumption, animals received 0.1 mL of a DTPA solution labeled with 18.5 mebequerel of ^99m^Tc by gavage. Four hours later, mice were anesthetized; their blood was collected, weighed, and placed in appropriate tubes for radioactivity determination. Blood radioactivity levels were determined using an automated gamma counter (Perkin Elmer Wallac Wizard 1470–020 Gamma Counter; PerkinElmer Inc., Waltham, MA, USA). Results obtained were compared with the standard dose and calculated as a percentage of the dose per gram of blood using the following equation:

% dose/g blood=(cpm in g of blood/cpm dose of standard)×100,

where cpm represents the counts of radioactivity per minute.

### Flow Cytometry Analyses

Following euthanasia, colon was harvested, and mesenteric lymph nodes were removed. Intestines were then cut open longitudinally and drawn through a pair of curved forceps while applying gentle pressure to remove intestinal contents. Tissues were cut into 2–4 cm fragments, washed twice to remove feces in calcium- and magnesium-free HBSS containing 2% FCS (at 4°C). Tissues were placed in 50 ml tubes and washed three times in HBSS containing 2% FCS at 4°C. Tissues were transferred to 25 cm^3^ tissue culture flasks and incubated at 37°C in HBSS containing 10% FCS, 0.2 mmol/l EDTA, 1 mmol/l DTT, 100 U/ml penicillin, and 100 µg/ml streptomycin. After 20 min, flasks were shaken vigorously for 30 s, and supernatant containing IEL was separated from the tissue fragments using a stainless steel sieve. To isolate lamina propria lymphocytes, the remaining tissue was washed three times with RPMI, and intestinal pieces were subsequently incubated for 30 min at 37°C in RPMI supplemented with 100 U/mL liberase (Roche). Cells were separated from tissue debris by purification through a 70 µm nylon filter. This step was repeated with a 40 µm nylon filter. Cell suspensions were adjusted to 10^6^ cells/ml.

Flow cytometry analysis for neutrophils was performed using the following antibodies: CD11c-APC, CD103-Pacific Blue, MHC (IA IE)-PE, Ly6C-Percp, Ly6G-FITC, CD45-APC-Cy7, and F4/80-PE-Cy7. For T lymphocyte analysis, the following antibodies were used: CD4-FITC, ROR-γt-PE, IL-23R-PercepCy5.5, CD45-APC-Cy7. For ILC analysis, the following antibodies were used: Pacific Blue: CD11b, CD11c, or FceR1a; BV421: CD16 or CD19; PERCP-CY5.5: CD8; PE/CY7: CD4; BV570: CD45; BV605: CD117; APC: CD127; F700: CD3; PE-CF594: ROR-γt; PE: t-bet; AF488: GATA 3. All antibodies were purchased from Biolegend (San Diego, CA, USA). Strategies used to analyze these cell populations are shown in Figures S1 and S2 in Supplementary Material. For surface antigen detection, cells were labeled with monoclonal antibodies for 30 min at 4°C. For intracellular labeling, a fixing/permeabilization kit (e-Bioscience, San Diego, CA, USA) was used after this step. Samples were then incubated for 30 min with a solution containing the appropriate antibodies. After washing with PBS containing 0.5% FBS, samples were fixed with 3% paraformaldehyde for 30 min, washed and stored in PBS at 4°C. Cells were acquired using a FACSCanto II cytometer (Becton Dickinson, East Rutherford, NJ, USA) and data was analyzed by FlowJo software (Tree Star, Ashland, OR, USA).

### Intestinal Tissue Preparation for Cytokine Measurement

Colon samples were weighted and homogenized in PBS containing 0.05% Tween-20, 0.1 mM phenylmethylsulphonyl fluoride, 0.1 mM benzethonium chloride, 10 mM EDTA, and 20 KIU Aprotinin A using a tissue homogenizer (100 mg tissue/ml buffer). Suspensions were centrifuged at 12.000g for 20 min at 4°C and the supernatants were transferred to microtubes. Supernatants were collected for cytokine assay levels of IFN-γ and IL-23 by capture ELISA. Briefly, plates were coated with purified monoclonal antibodies reactive with the cytokines IFN-γ and IL-23 (BD-Pharmingen, Franklin, NJ, USA) overnight at 4°C. In the following day, wells were washed, supernatants and standards were added, and plates were incubated overnight at 4°C. In the third day, biotinylated monoclonal antibodies against cytokines were added and plates were incubated for 2 h at room temperature. Color reaction was developed at room temperature with 100 μL/well orthophenylenediamine (1 mg/mL), 0.04% H_2_O_2_ substrate in sodium citrate buffer. Reaction was interrupted by the addition of 20 μL/well of 2 N H_2_SO_4_. Absorbance was measured at 492 nm by ELISA reader (Bio-Rad, Philadelphia, PA, USA).

### RNA Isolation, Reverse Transcription, and Quantitative Real-time PCR (qPCR)

Total RNA extraction from frozen colon of C57BL/6 mice that received either standard diet (control group) or HSD group for 3 weeks, 5 days, 3 days, 120 h, 48 h, 36 h, or 24 h and colon of mice that received a 50% NaCl solution by oral gavage and were euthanized after 90 min thereafter, was performed using TRIzol^®^ reagent (Invitrogen, USA), followed by treatment with RQ1 RNase-Free DNase (Promega, USA), and cDNA synthesis using RevertAid H Minus First Strand cDNA Synthesis Kit (Thermo Scientific, UK). qPCR assays were performed to evaluate the mRNA levels of *Sgk1* (serum/glucocorticoid-regulated kinase 1) gene. *Gapdh* (glyceraldehyde-3-phosphate dehydrogenase) gene was used for the normalization of the data. Primers were designed using Primer3 program, version 0.4.0 (http://frodo.wi.mit.edu/) and analyses of parameters such as GC content and formation of homo-dimer, hetero-dimer, and hairpins structures were performed using OligoAnalyzer 3.1 software (https://www.idtdna.com/calc/analyzer). The specificity of the primers was confirmed using Primer-BLAST program (http://www.ncbi.nlm.nih.gov/tools/primer-blast/), to ensure that the primers amplified only the coding region (CDS) of interest. The sequences of the forward and reverse primers used for amplification of *Sgk1* (GenBank: NM_001161847.2) and *Gapdh* (GenBank: GU214026.1) were 5′-CAAATCAACCTGGGTCCGTC-3′ (Sgk1-F), 5′-TCCAAAACTGCCCTTTCCG-3′ (Sgk1-R); 5′-CATCTTCCAGGAGCGAGACC-3′ (Gapdh-F), and 5′-GAAGGGGCGGAGATGATGAC-3′ (Gapdh-R). Each qPCR reaction contained 5 µL KAPA SYBR^®^ FAST qPCR Master Mix 2× (Kapa Biosystems, USA); 0.2 µL of ROX High 50×; 200 ng of cDNA; each forward and reverse primer at the optimized concentrations (0.4 µM (F)/0.4 μM (R) for Sgk1 and 0.3 µM (F)/0.3 μM (R) for Gapdh) and water up to a final volume of 10 µL. The reaction profile was an initial step of denaturation at 95°C for 3 min, followed by 35 cycles of denaturation at 95°C for 3 s and combined annealing and extension at 60°C for 30 s. A “no template control” was made in all the qPCR reactions for each pair of primers containing all the reagents except cDNA. Reaction specificity was confirmed with melting curves analysis and agarose gel electrophoresis experiments (*Sgk1* amplicon size = 87 bp; *Gapdh* amplicon size = 150 bp). Standard curves were generated with series of log dilutions of cDNA to calculate the amplification efficiency (*Sgk1*: Eff = 98.98%, *R*^2^ = 0.99; *Gapdh*: Eff = 96.28%, *R*^2^ = 0.99). The qPCR reactions were performed using ABI PRISM^®^ 7900HT Sequence Detection System (Applied Biosystems, UK), and the data were processed by SDS Software, version 2.4 (Applied Biosystems, UK). The calculation of gene expression was performed using the 2^−ΔCt^ method, where ΔC_t_ = C_t_ value of target gene − C_t_ value of reference gene.

### Colitis Induction

Dextran sodium sulfate-induced colitis was triggered by administration of 1% dextran sulfate sodium in the drinking water to C57BL/6 mice for 7 days (19). TNBS-induced colitis was triggered by administration of 2.5 mg TNBS in 35% alcohol intrarectally to BALB/c mice 7 days before euthanasia (19).

### Clinical Score and Histological Assessment of Colitis

Colons were excised and colonic inflammation assessed using a previously defined scoring system (20), which includes macroscopic features such as the presence or absence of adhesions, strictures, and diarrhea (diarrhea was defined as loose, watery stool). Samples of colon were fixed in formalin and processed for microscopic analysis. Hematoxylin-eosin-stained sections were blindly scored based on a semiquantitative scoring system that includes the main alterations observed: goblet cell depletion, muscular layer (erosion/ulceration) destruction, cellular infiltration, and edema; each parameter could receive 0–3 in the score index.

Colitis clinical severity (Disease Activity Index) were evaluated by a scoring system described previously (20) where the following features were graded: extent of destruction of normal mucosal architecture (0: normal; 1, 2, and 3: mild, moderate, and extensive damage, respectively), presence and degree of cellular infiltration (0: normal; 1, 2, and 3: mild, moderate, and transmural infiltration, respectively), extent of muscle thickening (0: normal; 1, 2, and 3: mild, moderate, and extensive thickening, respectively), presence or absence of crypt abscesses (0: absent; 1: present) and the presence or absence of goblet cell depletion (0: absent; 1: present). Scores for each feature were summed up to a maximum possible score of 11.

### Measurement of the Activity of Myeloperoxidase (MPO)

Samples were first homogenized in buffer solution (NaCl 0.1 M, Na_3_PO_4_ 0.02 M, and Na_2_EDTA 0.015 M) and then centrifuged. Supernatant was discarded and the pellet resuspended and centrifuged. Supernatant was discarded and the pellet resuspended in another buffer (Na_3_PO_4_ and 0.5% w/v HETAB) solution and then stored at room temperature in a ratio of 1.9 ml/100 mg. Samples were homogenized and half the volume was withdrawn for NAG enzyme activity measurement. The other half of the homogenate was used for the determination of MPO activity. From this step on, the samples received different treatments.

Evaluation of NAG activity was performed separately. Salina/Triton (Saline 0.9% and Triton X-100 0.1%) was added to the homogenate, samples were homogenized, and then centrifuged at 4°C for 10 min at 3000 RPM. Hundred microliters of the supernatant were collected and diluted in citrate phosphate buffer (0.1 M citric acid and 0.1 M Na_2_HPO_4_) for NAG measurement. In the microplate, 100 µL of each diluted sample was pipetted, and 100 µL of the substrate *p*-nitrophenyl-*N*-acetyl-β-d-glucosaminide diluted in phosphate citrate buffer was added to each well. Samples were incubated. Then, 100 µL of 0.2 M glycine buffer was added to the samples to stop the reaction. The absorbance was red at 400 nm. The mean of the values was obtained from each duplicate and was used to determine the activity of the enzyme.

### Statistical Analysis

All results were expressed as the mean ± SD of the mean. Significance of differences among groups was determined by either Student’s *t*-test or analysis of variance (ANOVA). Most of the data represent results from two or three independent experiments with five mice per group as indicated in the legend of figures. Means were considered statistically different when *p* < 0.05.

## Results

### High-Salt Diet Induced Augmented Arterial Pressure in Mice

**Figure 1:**
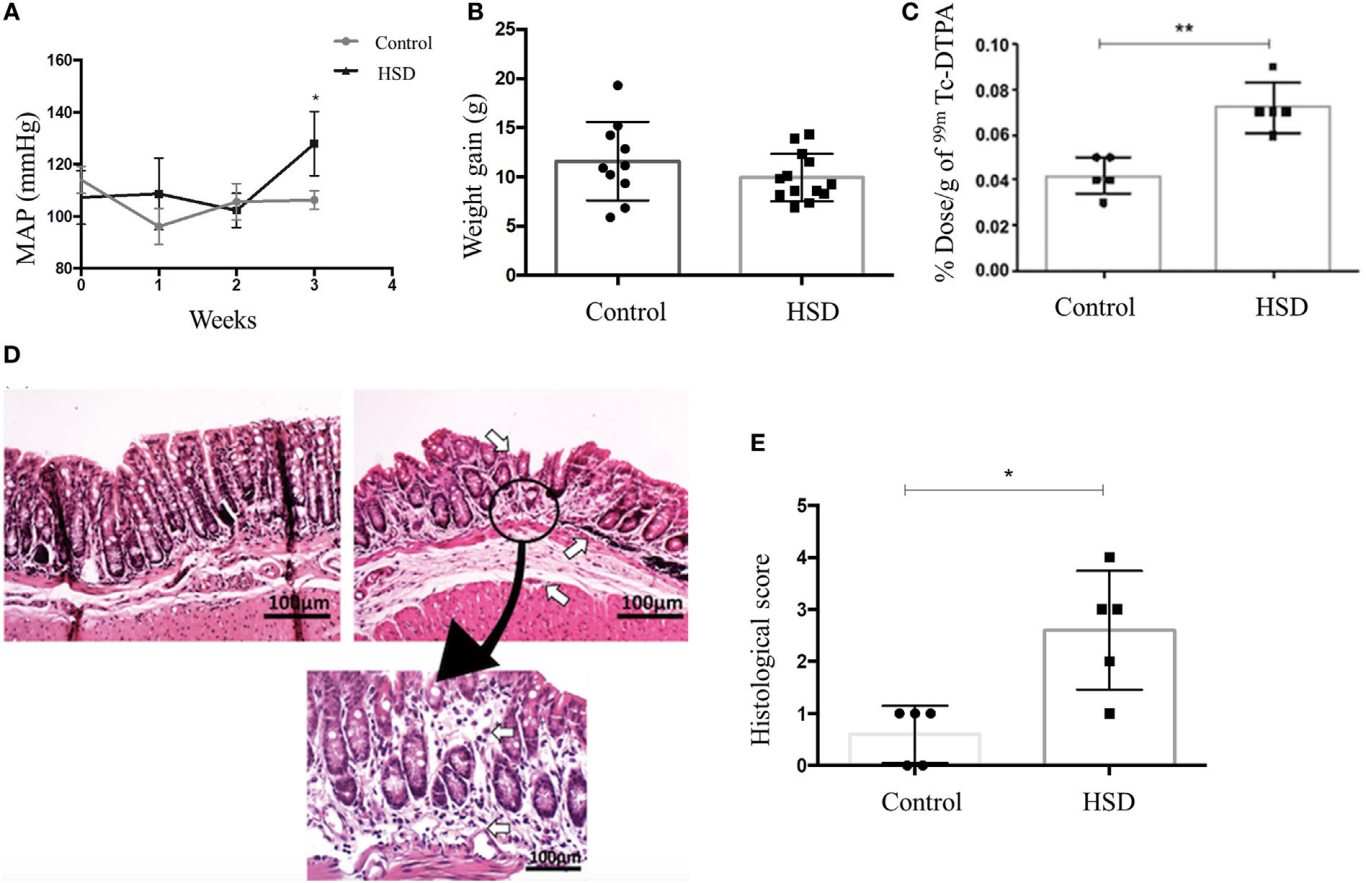
High-salt diet (HSD) consumption increased arterial pressure and intestinal permeability, and led to gut inflammation. **(A)** Blood volume measured in a plethysmograph to obtain the mean arterial pressure (MAP) of mice fed either standard diet (control) (*n* = 5–15) or HSD (*n* = 5–15) during 3 weeks. **(B)** Weight gain; **(C)** intestinal permeability; **(D)** representative histological image of colon of control mice (left), HSD fed mice (right) after 3 weeks of diet consumption (100× magnification). Area of gut inflammatory reaction of HSD fed mice colon is shown at 200× magnification; **(E)** Histological score comparing control and HSD fed mice after 3 weeks of diet consumption. Score represents the sum of edema, inflammation area, and erosion area. Graphs are representative of three independent experiments with five mice per group. Statistical analysis between control and HSD fed mice at the end time point of each experimental was performed using Student’s *t*-test for parametric data and Mann–Whitney for non-parametric data. Differences over time were evaluated using two-way analysis of variance test. *p < 0.05.

To confirm that 4% sodium chloride diet was able to increase the blood pressure, as expected for a HSD, we performed a plethysmography (a technique used to measure changes in body blood volume) in mice. After 3 weeks of HSD consumption, we observed an increase in mean arterial pressure (MAP) in mice fed HSD when compared to mice fed a standard diet (Figure 1A).

### High-Salt Diet Induced Histological Signs of Colonic Inflammation and Increase in Intestinal Permeability

Clinical signs of colitis such as weight loss, diarrhea, rectal bleeding, and reduced colon’s length were not observed in mice that received HSD when compared to mice fed standard diet (Figure 1B). Intestinal permeability was also measured as a more sensitive method to evaluate intestinal damage. Figure 1C shows that mice fed HSD had an increased intestinal permeability than control mice. Histological sections where obtained to access microscopic signs of inflammation. HSD-fed mice presented depletion of goblet cells, cellular infiltration in the colonic mucosa, erosion, ulceration, and destruction in *muscularis mucosa* layer, as well as edema in submucosal layer (Figure 1D). Histological scores were calculated and it showed inflammatory alterations in the HSD-fed mice (Figure 1E).

### High-Salt Diet Increased the Production of IL-23 and the Frequency of IL-23R^+^ CD4^+^ T Cells

**Figure 2:**
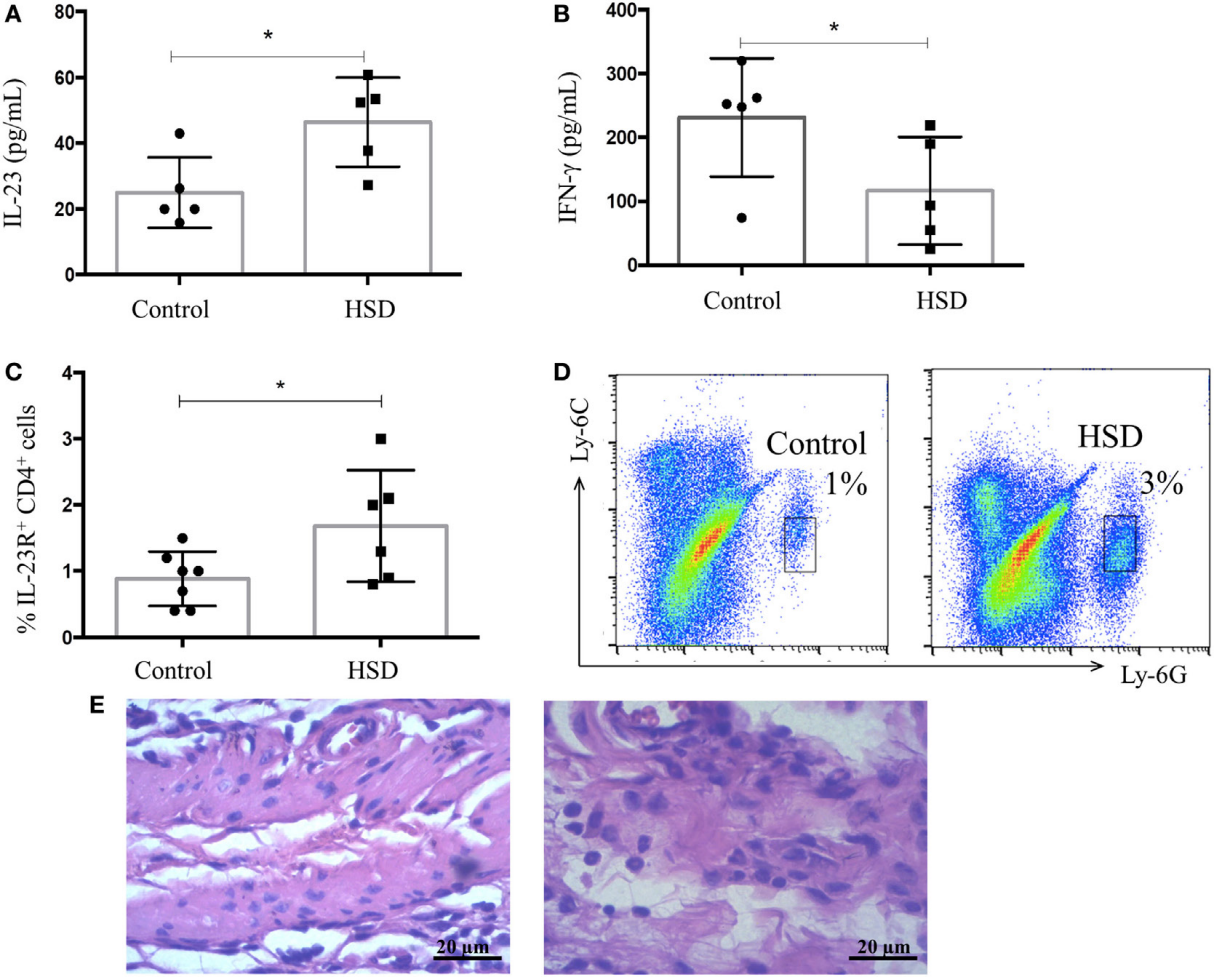
IL-23 concentration and frequency of neutrophils in colonic mucosa increased after high-salt diet (HSD) consumption. C57BL/6 mice received control diet (*n* = 5–15) or HSD (*n* = 5–15) for 3 weeks. **(A)** IL-23 concentration in colon extract of mice fed either control diet or HSD; and **(B)** IFN-γ concentration in colon extract of control diet- or HSD-fed mice measured by ELISA; **(C)** CD4^+^ IL-23R^+^ cell frequency obtained by flow cytometry; and **(D)** representative dot plot of neutrophils in colons of control group (left) and HSD group (right) analyzed by flow cytometry. **(E)** Representative histological image of colon of control (left) or HSD fed mice (right) after 3 weeks of diet consumption (200× magnification). Graphs are representative of two independent experiments with five mice per group. Statistical analysis between groups was performed using Student’s *t*-test. *p < 0.05.

Next, we investigated local factors that could lead to intestinal inflammation in HSD-fed mice. Colon extracts were examined for their content of IL-23, a cytokine involved in Th17 and type 3 innate lymphoid cell (ILC3) differentiation. IFN-γ, a typical Th1 cytokine, was also measured. HSD-fed mice presented increased levels of IL-23, but normal levels of IFN-γ in their colons when compared with the control group (Figures 2A,B, respectively). An increased frequency of CD4^+^ T cells expressing IL-23R^+^ in the colonic tissues (Figure 2C) was also observed. Th17 are known to secrete IL-17 and this inflammatory cytokine is involved in recruitment of neutrophils, ultimately leading to mucosal inflammation (21). To verify whether increased levels of IL-23 could be related to neutrophil infiltration in the colon, the frequency of these cells was analyzed by flow cytometry. Figure 2D shows an increased frequency of neutrophils in the colon of mice fed HSD for 3 weeks. This result was confirmed by the histological analysis of the colonic mucosa showing a significant infiltration of neutrophils in the colons of HSD-fed mice (Figure 2E).

### High-Salt Diet Led to Increase in the Frequency of ILC 3 in the Colon

**Figure 3:**
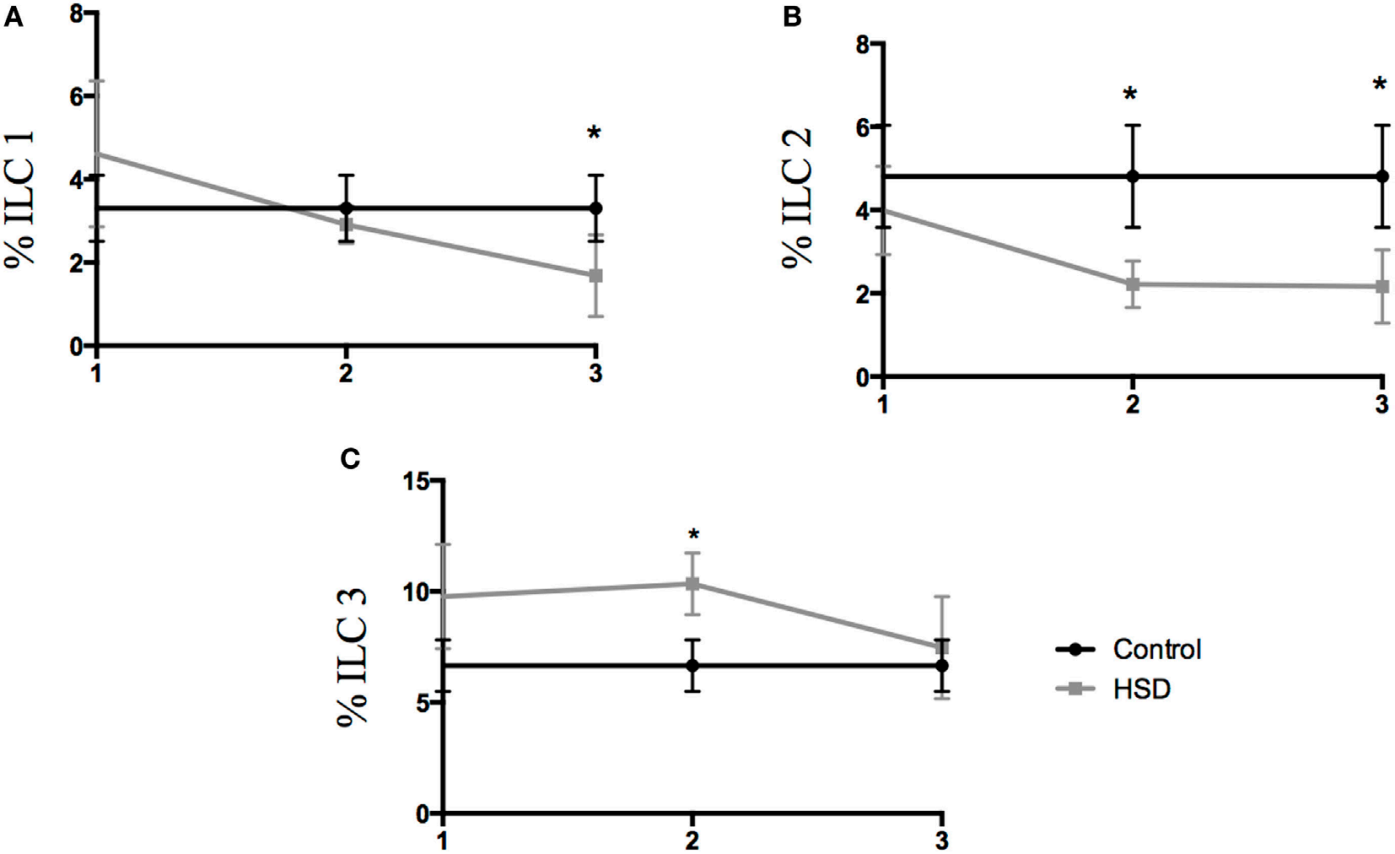
Frequency of innate lymphoid cell populations changed during high-salt diet (HSD) consumption. Frequencies of ILCs were analyzed by flow cytometry in the colonic *lamina propria* of mice that received either control diet (*n* = 5–10) or HSD (*n* = 5–10) during 1, 2, and 3 weeks. **(A)** ILC1 (Lin-t-bet^+^); **(B)** ILC2 (Lin-GATA3^+^); **(C)** ILC3 (Lin- RORγt^+^). Graphs are representative of two independent experiments with five mice per group. Statistical analysis between groups was performed using Student’s *t*-test. *p < 0.05.

Type 3 innate lymphoid cells (ILC3) are important cells for gut homeostasis but are also involved in the development of intestinal inflammatory diseases (16–18). Considering that IL-23 cytokine, that is elevated in our model, is closely associated with ILC3 differentiation, the frequency of colonic ILC populations where analyzed. ILC1 population was decreased in the colon after 3 weeks of HSD consumption (Figure 3A). ILC2 frequency also decreased after 2 and 3 weeks of HSD consumption (Figure 3B). However, ILC3 frequency was increased in the colon of mice fed HSD for 2 weeks (Figure 3C).

### High-Salt Diet Lead to Gut Inflammation in RAG^−/−^ Mice but Not in IL-17^−/−^ Mice

**Figure 4:**
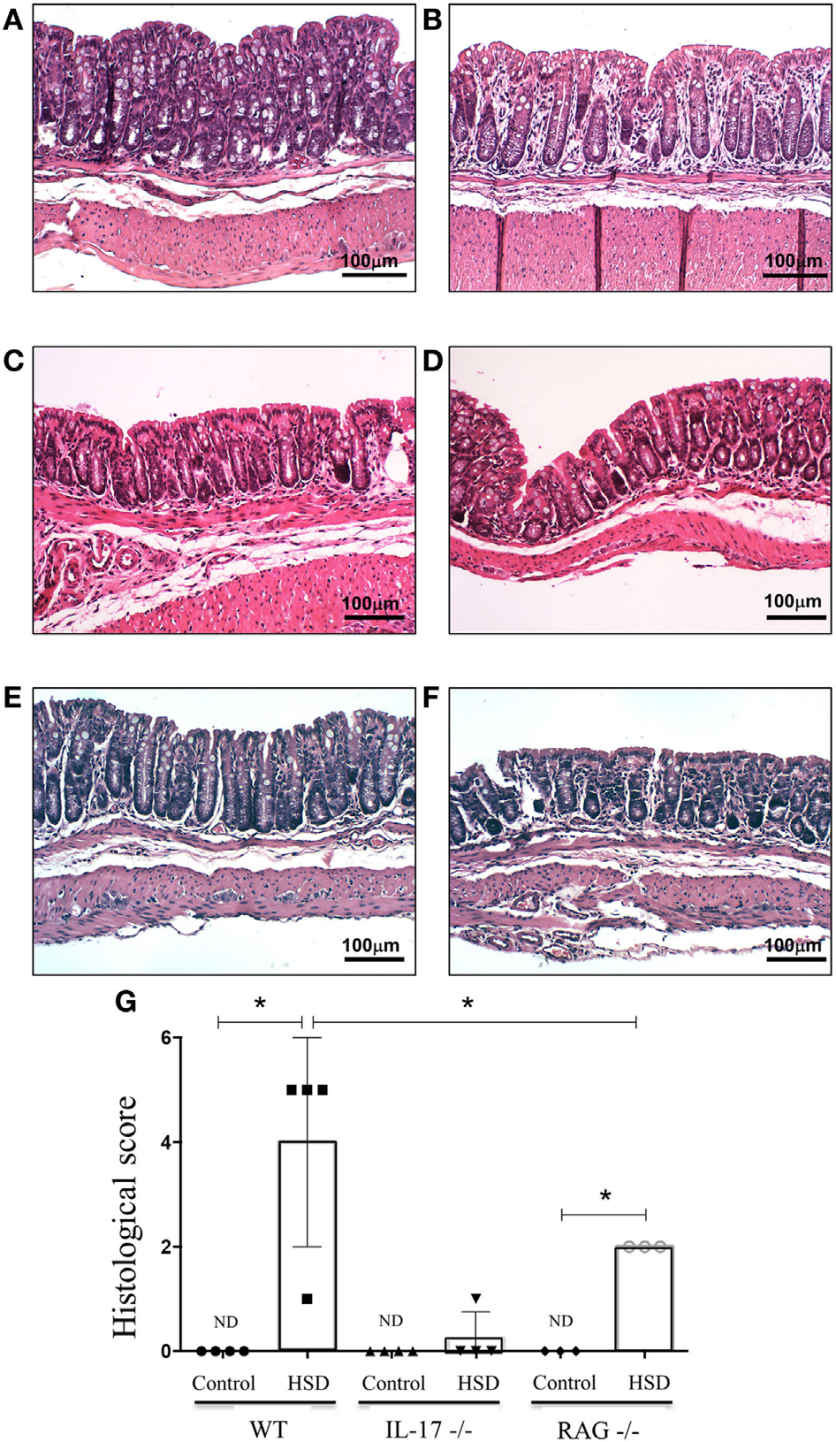
Consumption of high-salt diet (HSD) induced gut inflammation in RAG^−/−^ mice but not in IL-17^−/−^ mice. C57BL/6 wild type (WT), IL-17^−/−^ and RAG^−/−^ mice received either control diet (*n* = 3) or HSD (*n* = 3) for 3 weeks. For histological score, goblet cell depletion, muscular layer (erosion/ulceration) destruction, cellular infiltration, and edema were considered, each parameter received 0–3 in the score index. **(A,B)** Representative images of colon sections from WT mice fed either control diet or HSD, respectively; **(C,D)** representative histological image of colons of IL-17^−/−^ mice fed either control diet or fed HSD, respectively; **(E,F)** representative histological images of colon from RAG^−/−^ mice fed either control diet or HSD, respectively. **(G)** Histological score comparing IL-17^−/−^ and RAG^−/−^ with their respective WT littermates. Statistical analysis between groups was performed using Student’s *t*-test. *p < 005. Images are shown at 100× magnification.

Since we found that consumption of HSD was associated with IL-23 production and increased frequencies of ILC3 (important cells for IL-17 production), we examined next the role of IL-17 as a trigger of inflammation. Figures 4A,B show the histological section from colon of wild-type (WT) mice fed either control or HSD, respectively. To evaluate the role of IL-17 in the inflammatory process driven by HSD, we analyzed the histological scores of IL-17^−/−^ mice that received HSD diet for 3 weeks and no inflammatory infiltration in their colons was found (Figure 4D) suggesting the effects of HSD consumption were dependent on IL-17.

Since Figure 3C showed an augmented frequency of ILC3 in the colon of mice treated with HSD, we decided to investigate further if the colonic inflammation was dependent exclusively on T lymphocytes. To test this, RAG^−/−^ mice (that lack T and B lymphocytes) were fed HSD for 3 weeks. Figure 4E (RAG^−/−^ mice fed control diet) and Figure 4F (RAG^−/−^ mice fed HSD) show that HSD-fed RAG^−/−^ mice also developed histological signs of colonic inflammation when compared to their controls, although at a statistically significant lesser extent than HSD-fed WT mice (Figure 4G). These results suggest that the inflammation caused by HSD is dependent on IL-17 but only partially dependent on Th17 cells.

### High-Salt Consumption Lead to a Trend to Increase *sgk1* Expression in the Colon Shortly after NaCl Ingestion but Not Later

**Figure 5:**
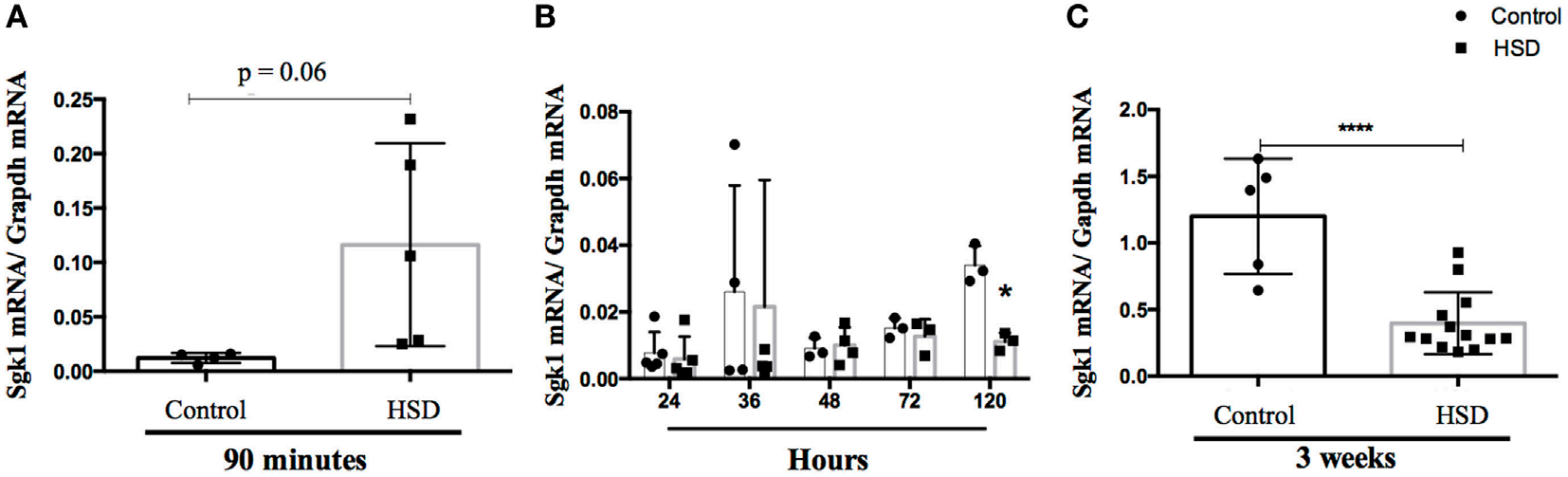
Consumption of high-salt diet (HSD) altered expression of SGK-1. mRNA was extracted from colon of C57BL/6 mice **(A)** 90 min after they received a 50% NaCl solution by gavage; **(B)** 24 h, 36 h, 48 h, 72 h, and 120 h after consumption of either control diet (*n* = 5–10) or HSD (*n* = 5–10) and **(C)** after 3 weeks of control diet (*n* = 5–10) or HSD consumption (*n* = 5–10). *Sgk-1* relative expression was quantified by quantitative real-time PCR in the colon extracts. Graphs are representative of three independent experiments with five mice per group. Statistical analysis between groups was performed using Student’s *t*-test. *p < 0.05.

Sgk1 is a kinase associated with Na^+^ absorption. An increase in its expression was previously associated with activation of Th17 cells and inflammation caused by high concentrations of salt in the western diet (5, 6). To verify whether there was any difference in *sgk1* expression in colon of HSD-fed mice, expression of this gene was evaluated either 90 min after oral administration (by gavage) of a solution containing high concentration of NaCl (50%) by gavage (Figure 5A) or 24, 36, 48, 72, and 120 h (Figure 5B) as well as 3 weeks (Figure 5C) after HSD consumption. There was no difference in the expression of *sgk1* up to 72 h post HSD consumption, but we could observe reduction in expression of sgk1 after 120 h and after 3 weeks of consumption. At an earlier time point, 90 min, there was a trend to increase in the expression of sgk1 (*p* < 0.06).

### High-Salt Diet Worsened DSS-Induced Colitis in C57BL/6 Mice

**Figure 6:**
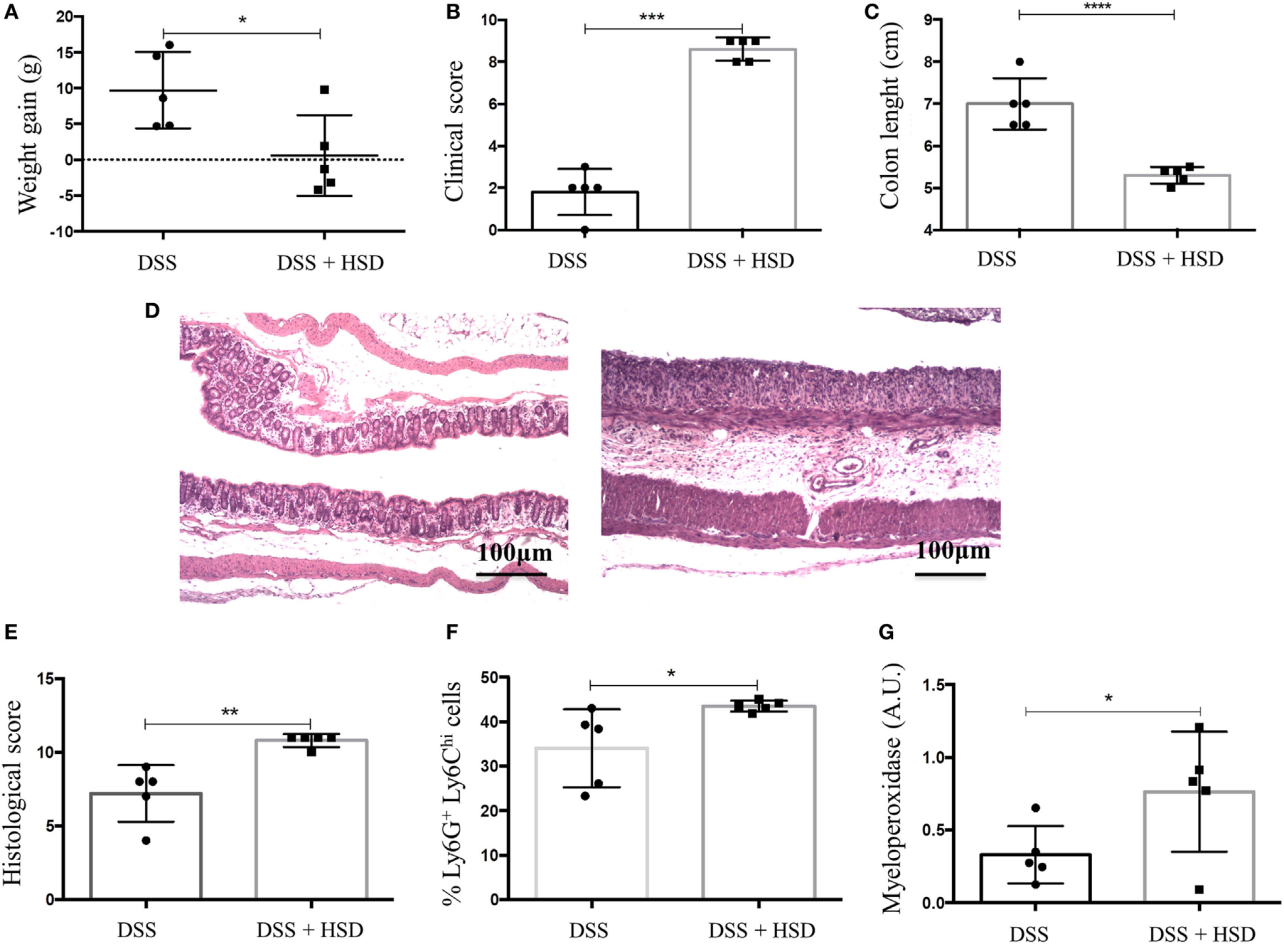
Consumption of high-salt diet (HSD) worsened dextran sodium sulfate (DSS)-induced colitis. C57BL/6 mice were fed either control diet or HSD for 3 weeks; mice received 1% DSS in drinking water in the last 7 days of the experiment. **(A)** Weight gain of DSS (*n* = 5–10) and DSS ^+^ HSD (*n* = 5–15) groups; **(B)** clinical scores of DSS and DSS^+^ HSD groups; **(C)** Colon lengths in centimeters of DSS and DSS^+^ HSD groups; **(D)** representative histological image from colon of DSS (left) and DSS^+^ HSD (right) groups; **(E)** histological score of DSS and DSS^+^ HSD groups; **(F)** Ly6G^+^Ly6C^Hi^ cell frequency in the colonic *lamina propria* measured by flow cytometry; **(G)** myeloperoxidase activity measured in colonic extract. For histological score, goblet cell depletion, muscular layer (erosion/ulceration) destruction, cellular infiltration, and edema were considered, each parameter received 0–3 in the score index. Graphs are representative of three independent experiments with five mice per group. Statistical analysis between groups was performed using Student’s *t* test. *p < 0.05.

After observing that HSD led to inflammation in the colon, we sought to investigate whether HSD could also worsen colitis inflammation. C57BL/6 mice were fed HSD for 3 weeks and in the last week they also received 1% DSS in the drinking water (19). Mice that received HSD did not show any weight gain (Figure 6A) and presented more severe clinical scores (Figure 6B) (diarrhea, weight loss, shortening of colon) and more severe shortening of the colon (Figure 6C) when compared to control mice challenged with DSS. Histological analysis (Figures 6D,E) also showed a more prominent inflammatory reaction in the colon of HSD-fed mice. Gut inflammation was associated with higher frequency of neutrophils (Figure 6F) and increased MPO activity (Figure 6G).

### High-Salt Diet Worsened TNBS-Induced Colitis in BALB/c Mice

**Figure 7:**
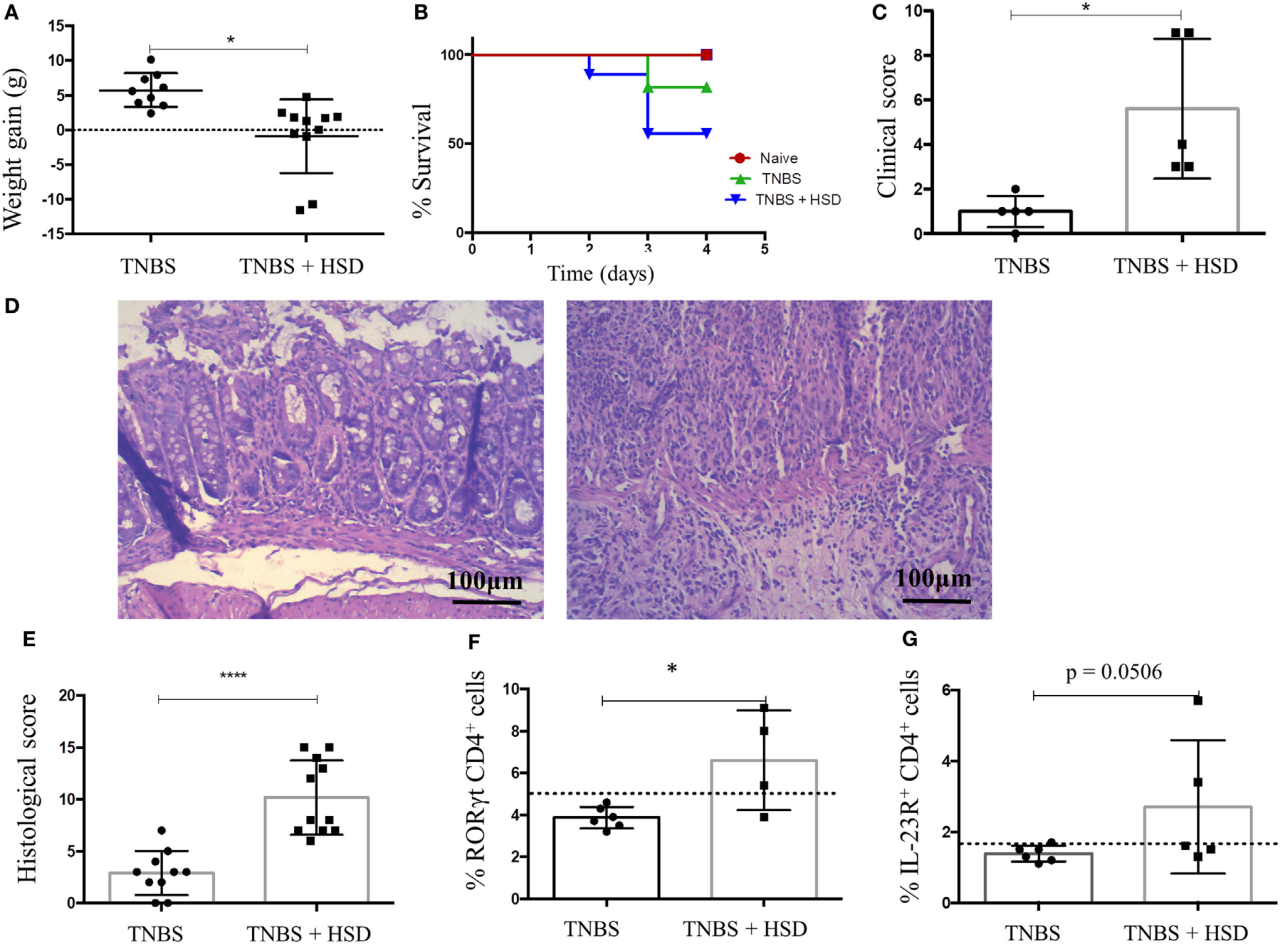
Consumption of high-salt diet (HSD) worsened 2,4,6-trinitrobenzenesulfonic acid (TNBS)-induced colitis in BALB/c mice. BALB/c mice were fed either control diet or HSD for 3 weeks. TNBS-induced colitis was induced by administration of 2.5 mg TNBS in 35% alcohol by intrarectal injections in mice 7 days before euthanasia. TNBS group (*n* = 5–15) received control diet for 21 days and TNBS^+^ HSD group (*n* = 5–15) received HSD throughout the experiment. **(A)** Weight gain during colitis; **(B)** survival curve; **(C)** clinical score; **(D)** representative histology images of TNBS (left) and TNBS^+^ HSD (right) groups; **(E)** histological score; **(F)** frequency of CD4^+^ RORγt^+^ cells in the colonic *lamina propria* of mice measured by flow cytometry; and **(G)** frequency of CD4^+^ IL-23R^+^ cells in the colonic *lamina propria* measured by flow cytometry. For histological score, goblet cell depletion, muscular layer (erosion/ulceration) destruction, cellular infiltration, and edema were considered; each parameter received 0–3 in the score index. Graphs are representative of three independent experiments with five mice per group. Statistical analysis between groups was performed using Student’s *t*-test. *p < 0.05.

2,4,6-trinitrobenzenesulfonic acid-induced colitis is a model of IBD triggered by Th1/Th17 cells (19). In order to evaluate the effects of HSD in TNBS-induced colitis, mice were fed HSD for 3 weeks, and in day 14 received TNBS diluted in alcohol by intrarectal injection. HSD-fed mice had a more pronounced weight loss (Figure 7A), decreased survival (Figure 7B), and increased clinical scores (Figure 7C) when compared to control TNBS-treated mice. Histological analyses also showed signs of a more severe gut inflammation (Figure 7D) and higher histological scores (Figure 7E) in HSD-fed mice.

Analysis of cells in the *lamina propria* of the colons from TNBS-treated mice showed an increased frequency of CD4^+^ ROR-γt^+^ T cells (Figure 7F) and CD4^+^ T cells expressing IL-23R (Figure 7G).

Of note, administration of diets rich in other salts such as magnesium and potassium chloride (data not shown) also resulted in worsening of DSS-induced colitis suggesting that pathways different from SGK1 may also lead to gut inflammation.

## Discussion

It is known that a high consumption of salt leads to lipid disorders, target kidney damage with hypertension, and decline in renal function (22), but little is known about the influence of HSD in immunological features. It has been demonstrated that increase in dietary NaCl concentration lead to Th17 cell differentiation and worsening of experimental autoimmune encephalomyelitis (EAE) (5, 6). In the present study, we sought to determine the consequences of consumption of HSD in the gut mucosa since this is the first tissue to contact all food components. Some recent studies demonstrated that consumption of HSD induces Th17 cells and exacerbates experimental colitis in mice thorough a SKG1 signaling pathway (12–14). However, none of these studies examined the direct damage caused by salt in the colonic mucosa without another source of insult. Moreover, they all proposed Th17 cells as the sole responsible for the IL-17 induced by high concentrations of sodium chloride.

We observed that consumption of a diet supplemented with 4% NaCl for 3 weeks led to a histologically identifiable inflammation in the colonic mucosa of mice with depletion of goblet cells, cellular infiltration, erosion, ulceration, and destruction in *muscularis mucosa* layer, as well as edema in submucosal layer. Other typical signs of mucosal inflammation such as increase in intestinal permeability were also present.

Inflammatory events triggered by HSD included production of high levels of IL-23, but not INF-γ, increased frequency of CD4^+^ IL-23R^+^ T cells and neutrophils in the colonic mucosa suggesting that the IL-17 axis was activated. Indeed, no histological sign of gut inflammation was detected in IL-17^−/−^ mice confirming that the HSD effect was dependent on this cytokine. Another recent study by Wei and coworkers also showed that exacerbation of colitis by HSD was dependent on IL-17 production (12).

Moreover, the inflammatory reaction induced by HSD consumption involved significant increase in the frequency of type 3 innate lymphoid cells (ILC3) in the colonic *lamina propria*. The decrease observed in ILC1 and ILC2 frequencies may have occurred as a compensatory balance after ILC3 increase. It has been reported that ILC3 have homologous functions to Th17 and that these cells are very abundant in the gut mucosa. They are also shown to be involved in intestinal homeostasis as they are producers of IL-22 and/or IL-17 in response to IL-23 and IL-1β (18). To examine whether the inflammation observed in HSD-fed mice was dependent exclusively on T cells or whether innate cells such as ILC3 could also be involved, we used RAG^−/−^ mice. These mice developed a histologically identifiable inflammation in their colonic mucosa after 3 weeks of HSD consumption indicating that T cells are not necessary for triggering gut inflammation. Indeed, the role of ILCs in the pathogenesis of IBDs has been widely acknowledged, and these cells are considered as potential therapeutic targets (15, 16, 18). The role of ILCs is also described in experimental models of intestinal inflammation associated with epithelium damage and IL-1β secretion by intestinal epithelial cells. Excessive amounts of sodium chloride in the diet may act directly in intestinal epithelial cells stimulating IL-1β secretion. This cytokine has been reported to promote innate immune pathology in *Helicobacter hepaticus*-triggered intestinal inflammation by augmenting the recruitment of granulocytes and the accumulation as well as activation of innate lymphoid cells (ILCs). In this study, IL-1β promoted Th17 responses from both CD4^+^ Th17 cells and ILC3 in the intestine, and a synergistic interaction between IL-1β and IL-23 signals sustained innate and adaptive inflammatory responses in the gut (23). In our case, the participation of Th17 cells in the effects of HSD is clear since the histological scores of RAG^−/−^ mice were lower than the ones of WT mice. However, our results also show that the IL-17-dependent gut inflammation occurred in the absence of lymphocytes. There was a significant difference in histological scores between WT and RAG^−/−^ mice suggesting other cells are involved. Therefore, it is likely that both cell types (Th17 cells and ILC3) participated in triggering inflammation upon high-salt exposure.

IL-17 induces the release of chemokines and other chemoattractants from epithelial and endothelial cells that enhance the inflammatory response through the recruitment of neutrophils (21). Protective effects of IL-17, in IBD, for example, are also reported. This dichotomy was recently explained by the observation that IL-17 promotes epithelial barrier function in the absence of IL-23 (24, 25). Hence, IL-23-independent IL-17 production is protective, whereas IL-23-dependent IL-17 production might be deleterious. In our study, gut inflammation caused by HSD was associated with increase in IL-23 production and in the frequency of CD4^+^ T cells bearing IL-23R, suggesting that these cells have an inflammatory role in colon of mice. Of note, no alteration was observed in the frequencies of CD4^+^ CD25^+^ Foxp3^+^ T cells in the colonic mucosa of HSD-fed mice (data not shown).

It has been described that Th17 cells are induced by HSD through SGK1 signaling pathway (5, 6). We tested SGK1 expression 90 min after oral administration of NaCl-containing solution and at several time points after HSD consumption (24, 36, 48, 72, 120 h, and 3 weeks after the beginning of feeding). We found no increase in sgk1 expression up to 72 h, despite a tendency for elevation in its expression at an earlier time point (90 min) and a decrease after 3 weeks of diet consumption. Since SGK-1 is an early gene, the expression of which increases to a steady state within hours (26), this early elevation right after high-salt ingestion and the decrease of its expression to normal levels after HSD consumption suggests that SGK1 and other signaling pathways may participate in the inflammatory effect triggered by high salt, especially when ILCs are involved. Moreover, SGK-1 expression could be increased in the first minutes after high-salt concentration and this elevation might be overturned by a compensatory mechanism later on. Results on SGK-1 involvement upon HSD consumption are ambiguous in other studies. In studies using rats, the authors detected a reduction in SGK-1 expression upon high-salt intake. In another study by the same group, rats fed a HSD for 5 weeks showed no change in SGK1 expression (26, 27). SGK-1 was decreased in the colon after adrenalectomy, but remained unchanged after sodium depletion or aldosterone injection (28), while other studies reported an increased induction upon these very same interventions (29, 30).

Our data also showed that consumption of HSD was also able to worsen colitis induced by both DSS and TNBS in C57BL/6 and BALB/c, respectively. Further investigation revealed that colitis exacerbation was associated with differentiation of Th17 cells, which occurred with an increase in the expression of IL-23R and the transcription factor RORγt by CD4^+^ T cells, as previously described by others (12, 14). It is plausible that the initial inflammatory event triggered by HSD depended on both ILC3 and Th17 cells. This early insult would predispose the colonic mucosa to increased production of IL-17 by a secondary trigger such as DSS or TNBS.

Of note, consumption of diets containing high concentrations of other salts such as potassium chloride also exacerbated DSS-induced colitis (Figure S3 in Supplementary Material) suggesting that sodium chloride and SGK1 activation may not be the only triggers of inflammation by HSDs.

Interestingly, a recent study showed that high-salt intake induces Th17 activation and worsening of EAE by affecting the gut microbiome in mice, particularly by depleting *Lactobacillus murinus* (31). Therefore, more than one mechanism seems to be triggered by high concentrations of salts resulting in inflammatory IL-17-dependent events. We cannot rule out the possibility that a change in the gut microbiota by HSD consumption also acted to induce gut inflammation and exacerbation of colitis.

Our findings indicate that, in addition to worsening colitis, HSD consumption itself was able to cause inflammation in the colonic mucosa of mice. The alterations induced by HSD were associated with increased frequencies of IL-23R^+^ CD4^+^ T cells and type 3 innate lymphoid cells (ILC3) and were dependent on IL-17 but not exclusively on T cells.

## Ethics Statement

All procedures were approved by the local ethical committee for animal research (CEUA-UFMG, Brazil, 50/2014). Experiments were performed in accordance with guidelines and regulation established by Conselho Nacional de Controle de Experimentação Animal (CONCEA), Brazil.

## Author Contributions

SA performed the experiments, discussed the results, and wrote the manuscript; MM helped performing the experiments and writing the manuscript; MG helped with colitis experiments; HS and CQ supervised and discussed the results of experiments with ILCs; PC performed SGK1 expression analysis; DC supervised and discussed the results of histological analysis; GF and AJF performed and discussed the results of the blood pressure analysis; PB performed the measurement of intestinal permeability with the supervision of VC; TM co-supervised the experiments, helped with discussion of the results and wrote the manuscript; AMCF supervised all the experiments, discussed the results, and wrote the manuscript.

## Conflict of Interest Statement

All authors declare no conflict of interest in the results presented. This study is not published in any other journal.

## Supplementary Material

Figure S1 Flow cytometry strategy analyses for innate lymphoid cells. The lymphocyte gate was defined considering SSC-height and FCS-height; within the lymphocyte gate, Lin^−^ CD45^+^ cells were selected, then CD3^+^ cells, next CD45^+^ CD127^+^ cells and finally each ILC was defined considering the expression of different transcriptional factors: (a) ILC1 were t-bet^+^ cells; (b) ILC2 were GATA3^+^ cells and (c) ILC3 were RORγt^+^ cells. Lin^–^ = CD3, CD4, CD8, CD16, CD19, CD11c, FceR1a. Pacific Blue-labeled anti-CD11b, BV421-labeled anti-CD16, BV421-labeled anti-CD19, Pacific Blue-labeled ant-CD11c, Pacific Blue-labeled anti-FceR1a, PERCP-Cy5.5-labeled anti-CD8, PE-Cy7-labeled anti-CD4, BV570-labeled anti-CD45, BV605-labeled anti-CD117, APC-labeled anti-CD127, AF700-labeled anti-CD3, PE-CF594-labeled anti-RORγt, PE-labeled anti-t-Bet, and AF488-labeled anti-GATA3 were used. Samples were acquired in FACSfortessa and data analyzed in FlowJo software 7.0.

Figure S2 Flow cytometry strategy analyses for neutrophils. First, the granulocyte gate was defined considering FCS-height and SSC-height, then CD11b^+^ cells were selected and F4/80^+^ cells excluded. Finally, Ly6C^Hi^ Ly6G^Int-Low^ analyzed as neutrophils. PERCP-labeled anti-CD11b, APC-labeled anti-F4/80, PE-labeled anti-Ly6C, and FITC-labeled anti-Ly6G antibodies were used. Samples were acquired in FACScan and data were analyzed by FlowJo software 7.0.

Figure S3 Hemoglobin and Clinical Score in dextran sodium sulfate (DSS)-induced colitis in mice fed NaCl-supplemented diet and KCl-supplemented diet. C57BL/6 mice were divided into six groups: control group received control diet and water during 3 weeks of experiment; high-salt diet (HSD) group received HSD containing 4% NaCl for 3 weeks; KCl group received diet containing 4% KCl during 3 weeks; DSS group received control diet and DSS 1% in the drinking water in the last 7 days of dietary consumption; HSD^+^ DSS received HSD with 4% of NaCl for 3 weeks and DSS 1% in the last 7 days of the experiment; HSD^+^ DSS received HSD with 4% KCl for 3 weeks of experiment and DSS 1% in the last 7 days of the experiment. **(A)** Hemoglobin concentration in peripheral blood and **(B)** Clinical score of colitis. Statistical analysis among groups was performed using one-way ANOVA. **p* < 0.05.
