# Supplementary figures and images for: High-Salt Diet Induces IL-17-Dependent Gut Inflammation and Exacerbates Colitis in Mice
Source: Front Immunol. 2018 Jan 15;8:1969. doi: 10.3389/fimmu.2017.01969 (PMC5775230; doi:10.3389/fimmu.2017.01969)

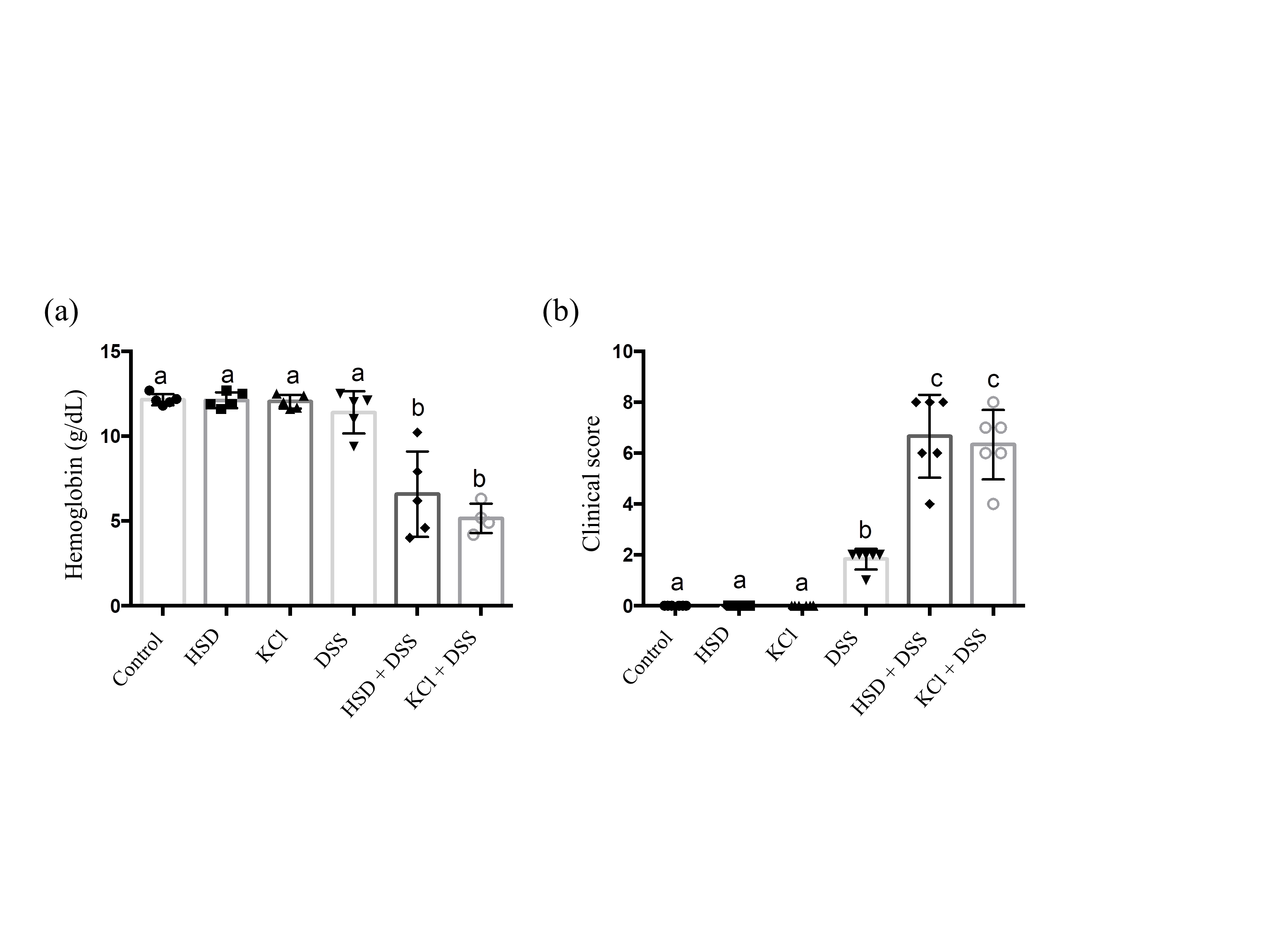

Supplement: Figure S1 — Flow cytometry strategy analyses for innate lymphoid cells. The lymphocyte gate was defined considering SSC-height and FCS-height; within the lymphocyte gate, Lin− CD45+ cells were selected, then CD3+ cells, next CD45+ CD127+ cells and finally each ILC was defined considering the expression of different transcriptional factors: (a) ILC1 were t-bet+ cells; (b) ILC2 were GATA3+ cells and (c) ILC3 were RORγt+ cells. Lin– = CD3, CD4, CD8, CD16, CD19, CD11c, FceR1a. Pacific Blue-labeled anti-CD11b, BV421-labeled anti-CD16, BV421-labeled anti-CD19, Pacific Blue-labeled ant-CD11c, Pacific Blue-labeled anti-FceR1a, PERCP-Cy5.5-labeled anti-CD8, PE-Cy7-labeled anti-CD4, BV570-labeled anti-CD45, BV605-labeled anti-CD117, APC-labeled anti-CD127, AF700-labeled anti-CD3, PE-CF594-labeled anti-RORγt, PE-labeled anti-t-Bet, and AF488-labeled anti-GATA3 were used. Samples were acquired in FACSfortessa and data analyzed in FlowJo software 7.0. [file Image_1.TIF]

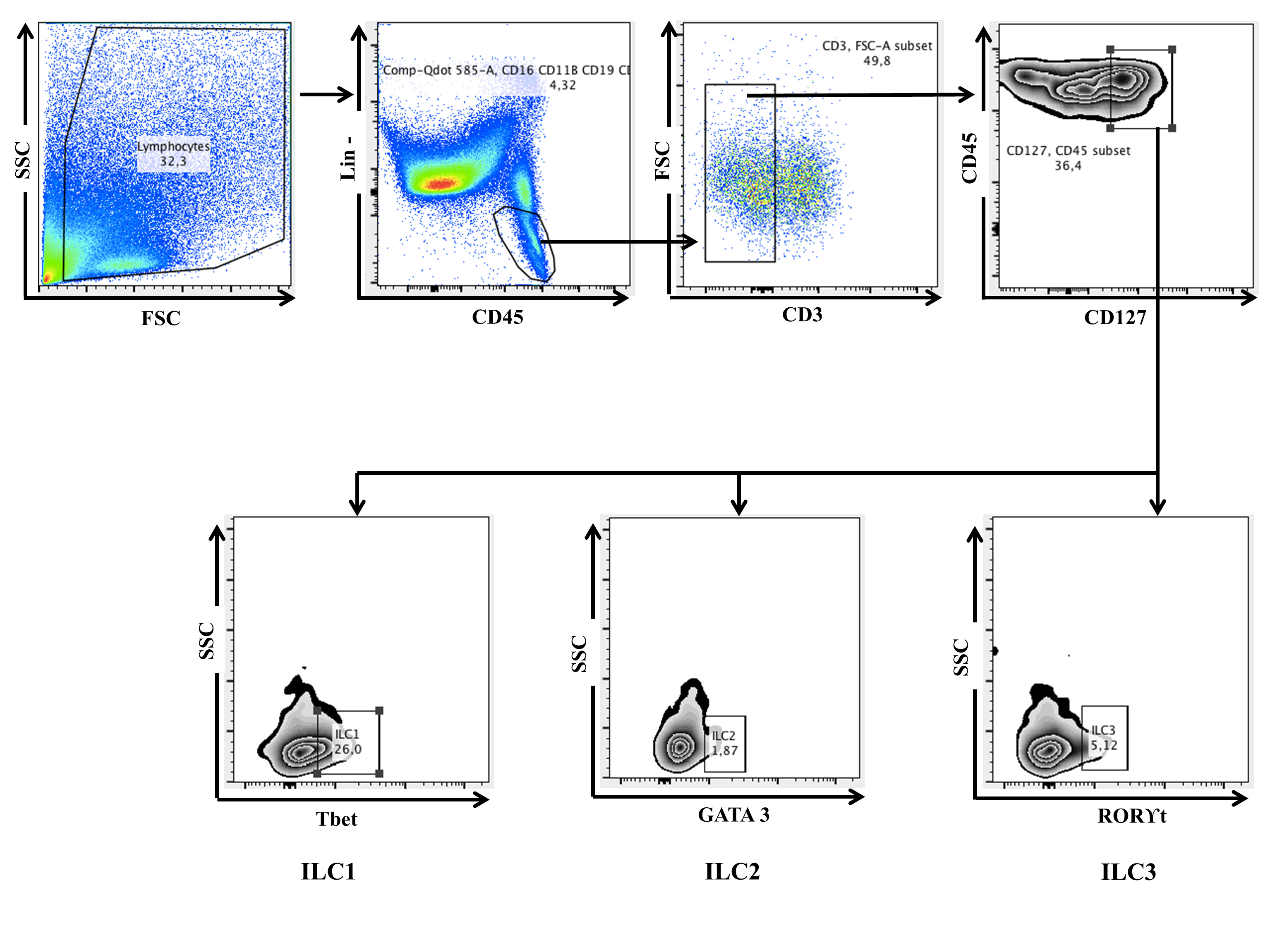

Supplement: Figure S2 — Flow cytometry strategy analyses for neutrophils. First, the granulocyte gate was defined considering FCS-height and SSC-height, then CD11b+ cells were selected and F4/80+ cells excluded. Finally, Ly6CHi Ly6GInt-Low analyzed as neutrophils. PERCP-labeled anti-CD11b, APC-labeled anti-F4/80, PE-labeled anti-Ly6C, and FITC-labeled anti-Ly6G antibodies were used. Samples were acquired in FACScan and data were analyzed by FlowJo software 7.0. [file Image_2.TIF]

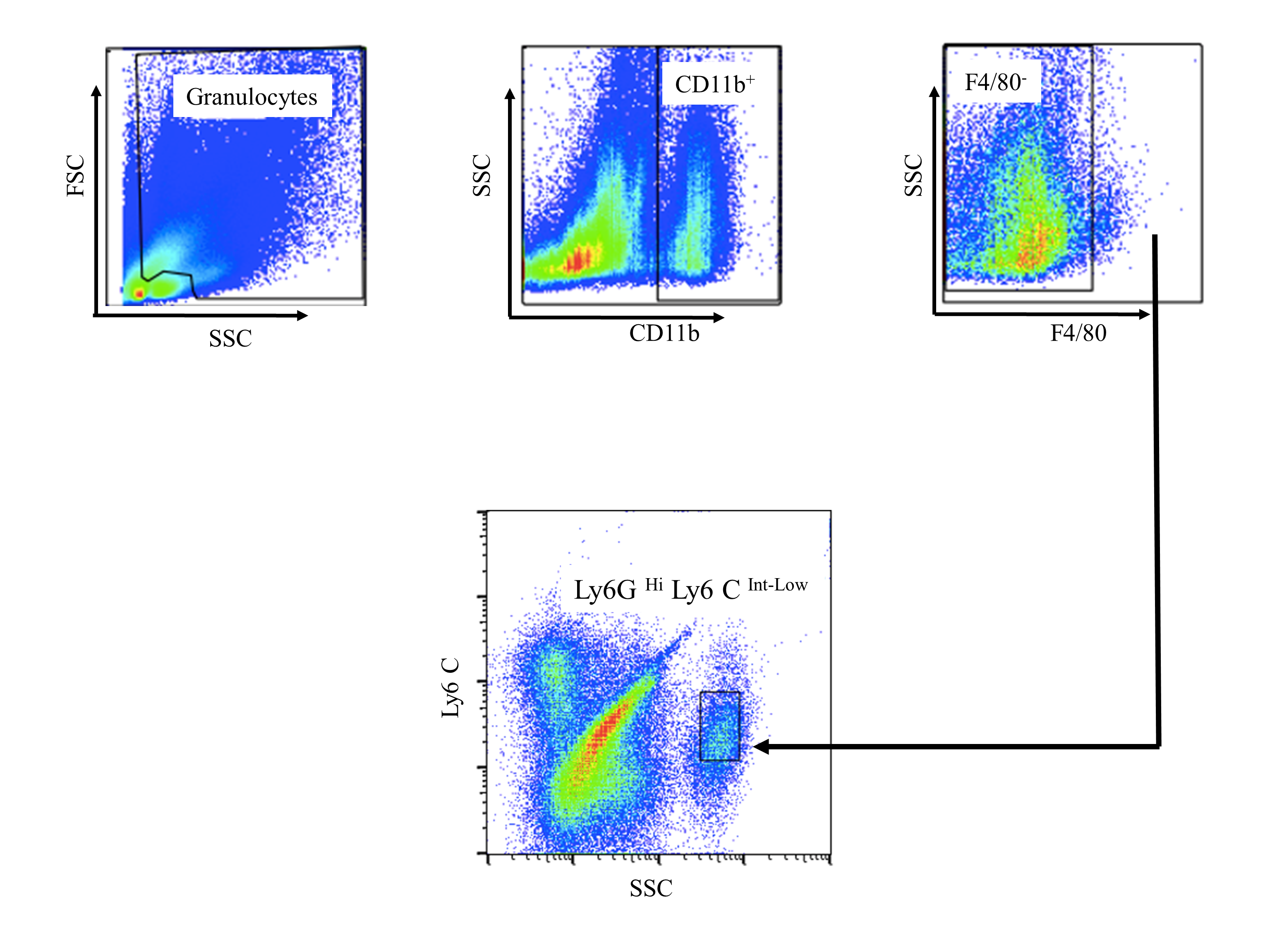

Supplement: Figure S3 — Hemoglobin and Clinical Score in dextran sodium sulfate (DSS)-induced colitis in mice fed NaCl-supplemented diet and KCl-supplemented diet. C57BL/6 mice were divided into six groups: control group received control diet and water during 3 weeks of experiment; high-salt diet (HSD) group received HSD containing 4% NaCl for 3 weeks; KCl group received diet containing 4% KCl during 3 weeks; DSS group received control diet and DSS 1% in the drinking water in the last 7 days of dietary consumption; HSD+ DSS received HSD with 4% of NaCl for 3 weeks and DSS 1% in the last 7 days of the experiment; HSD+ DSS received HSD with 4% KCl for 3 weeks of experiment and DSS 1% in the last 7 days of the experiment. (A) Hemoglobin concentration in peripheral blood and (B) Clinical score of colitis. Statistical analysis among groups was performed using one-way ANOVA. *p < 0.05. [file Image_3.TIF]
